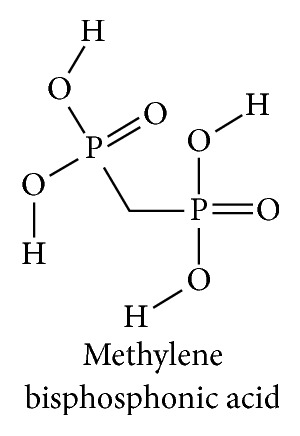# An Evaluative History of Bisphosphonate Drugs: Dual Physiologic Effects of Pyrophosphate as Inspiration for a Novel Pharmaceutical Class

**DOI:** 10.1155/2016/1426279

**Published:** 2016-10-05

**Authors:** W. Banks Hinshaw, Allyn F. DeLong

**Affiliations:** ^1^Markle & Hinshaw Gynecology and Harris Regional Hospital, 7190 Ellijay Road, Franklin, NC 28734, USA; ^2^Eli Lilly and The Indiana EPA, 10113 Partridge Place, Carmel, IN 56033, USA

## Abstract

The documented history of the development of the bisphosphonate drugs is reviewed in sufficient detail to permit independent evaluation of the consistency of the conclusions reached from the available data. The evidence developed during the early interval of these studies 1960–1975 was sufficient to establish that pyrophosphate shares the subsequently established dual bisphosphonate characteristics of bone resorption inhibition and prevention of tissue mineralization.

## 1. Introduction

The persistent controversy [1–3] swirling about the safety and efficacy of the bisphosphonate (BP) class of medications has prompted us to provide an objective review of the early development of these drugs. Understanding the original studies leading to the introduction of BPs for indications ranging from the alleviation of pain from metastatic disease to the treatment or prevention of osteoporosis is fundamental for the comprehension of the appropriate place of these medications in the modern pharmacopeia. Relatively complete historical coverage is possible because of the small number of research groups participating in the early discoveries. This began with the discovery of some physiologic properties of pyrophosphate (PPi, representing the ionized form of pyrophosphoric acid at physiologic pH). A recent review [4] of PPi metabolism emphasized the well-known tissue mineralization inhibition effects of this natural product but ignored the existence of the evidence showing antiresorptive properties. Thus, extending the legacy position [5] that antiresorptive properties could not be demonstrated, it seems to have been forgotten that this lack of evidence was originally characterized as uncertain and confounded by issues of substrate stability. The legacy investigators fully described the experimental barriers to the potential acquisition of such evidence. The recent review took a far more doctrinaire position: “pyrophosphate does not inhibit bone resorption, whereas this is the key pharmacological action of BPs when used to treat clinical disorders characterized by excessive resorption” [4]. This characterization arguably can and probably did result in misleading conclusions in situations where understanding the antiresorptive effect of PPi is pivotal. The purpose of our review is to describe the details and assess the validity of the conclusions drawn from the specific experimental results, conclusions which may have encouraged this doctrinaire opinion. We will set the record straight regarding some long neglected observations [6] by one of us (AFD) working in the lab of the respected American physiologist Rasmussen 45 years earlier. We shall also report additional legacy research consistent with PPi as antiresorptive.

## 2. Materials and Methods

We have reviewed, critically abstracted, and reported here the pertinent publications from the laboratories of Neuman at the University of Rochester, Fleisch, at Bern, and subsequently at Davos, Marion Francis at Proctor & Gamble Research in Ohio, and Russell at Davos and later at Oxford. Some publications have been omitted from the review if deemed not germane to the issue of pyrophosphate physiology. The studies of Rasmussen at the University of Pennsylvania and Orimo at the University of Tokyo will be shown to have augmented the incomplete conclusions reached by the other workers. Our review is limited to the earliest phases of this research between 1960 and 1975.

## 3. Results and Discussion

### 3.1. Initial* In Vitro* Discoveries

Every BP FDA-approved label contains the information that BPs “are synthetic analogs of PPi that bind to the hydroxyapatite found in bone.” In his comprehensive review of the developments of the drug class through 2000, Fleisch acknowledged that these PPi analogs exhibit dual dose- and structure-dependent characteristics [7]. BP “effects consist mainly of an inhibition of bone resorption and, when given in large amounts, an inhibition of ectopic and normal calcification. While the latter effect is the consequence of a physical chemical inhibition of calcium phosphate crystal formation, the former is due to a cellular effect….”

The path to the discovery of the BP class of oral and intravenous medications can be followed back to studies [8] in the laboratories of Neuman in the late 1950s where it was discovered that physiologic fluids contained a substance which inhibited the precipitation of calcium phosphate from unstable supersaturated solutions known otherwise to crystallize when seeded with several recognized collagen-derived moieties [9, 10]. This entity was preliminarily characterized as a polyphosphate whose removal by hydrolysis with phosphatase was required to permit this crystallization, exemplified in nature by the mineralization of tissues such as bone.

Neuman's work was continued by Fleisch on his return to Switzerland after a postdoctoral stint in the Neuman laboratories. Fleisch confirmed [11] that PPi in urine was a potent inhibitor of precipitation of calcium phosphate. He also discovered [12] using simple physical chemistry preparations that the effects of PPi were twofold. By 1966, Fleisch, now joined in Switzerland by Russell, was able to report that hydroxyapatite (HAp) crystals (hydrated calcium phosphate), when coated with PPi, were measurably less soluble at physiologic pH than uncoated HAp. He speculated that “if osteoporosis represents a disease of bone dissolution, it would be interesting to know whether pyrophosphate or other polyphosphates could be concerned either in the causation or the* treatment* [our emphasis] of the disease”. It is possible to date the beginning of the development of the BP class of medications from this statement. Fleisch and Russell had discovered a physical chemical decrease in the solubility of solid phase HAp when the crystals had been exposed to PPi. Soon this led to the examination of potential PPi analogs.

### 3.2. Early* Ex Vivo* Studies

Investigation of this effect in* ex vivo* experiments using cultured living embryonic chick femurs followed. As doubtless anticipated from the preceding work, PPi inhibited mineralization of this growing tissue at 4 and 16 *µ*gms P/mL but was also found to* increase* net bone mineral “deposition” [13] at 1 *µ*gm P/mL. The potency of this effect was high, despite being disparaged in the abstract as “slight.” The net increase in mineral content by two measurement techniques was ~45% more than control. Thus, by 1966, new discoveries were revealing a dual role for PPi in living tissue. These roles were assumed at the time to represent the same non-cell-mediated roles observed* in vitro*. This hypothesis was soon discarded based on a series of experiments described below. Changes in research emphasis due to practical pharmaceutical considerations, including route of administration and agent stability, now seem to have been confounded with a denial of the evidence the group had already published that PPi could also act to preserve and increase bone mineral at low concentrations.

**Figure 1:**
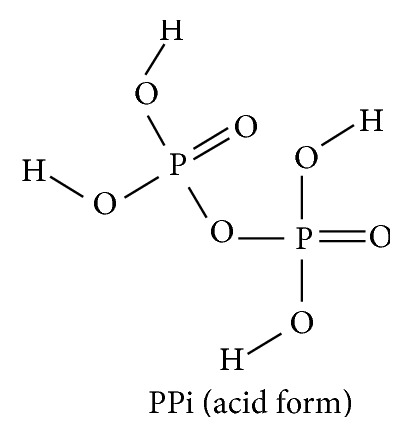


In subsequent experiments comparison of the mineral “deposition,” effects of PPi to several hydrolytically stable orally effective bisphosphonic acids led to favoring development of the latter from that point forward. Figures 1 and 2 show the structural similarity between PPi and the simplest bisphosphonic acid.

A short preliminary report in 1968 introduced the next phase of the investigation. Bisphosphonic acids were shown to inhibit aortic calcification and also to inhibit, as it was now termed, bone resorption. PPi antiresorptive effects were precluded. “A further* difference* (our emphasis) from pyrophosphate is that the diphosphonates prevent bone resorption.” [14]. But in this report, the* potential* of PPi as antiresorptive was at least acknowledged. “Since the diphosphonates are close structural analogues of pyrophosphate these results strengthen our earlier hypothesis that pyrophosphate could inhibit bone resorption* in vivo* and that its apparent failure to do so when given exogenously may be due to its local enzymic destruction.” This paper introduced mouse calvarial experiments (see below) but did not report any data for PPi effects.

The effects of PPi were further investigated, but the clue from the embryonic chick femur experiments that PPi, at very low levels, might prevent mineral loss as well as inhibiting mineral gain of bone at higher concentrations* in vivo* was ignored [15] by Russell and Fleisch 2 years later when they published additional data from the previously mentioned* ex vivo* preparation using chick hemicalvaria. For some reason, measurements dosing with PPi at any level were not reported in this* ex vivo* model, perhaps due to a belief [16] that the BPs represented a more fruitful avenue of exploration. The BPs structurally analogous to PPi inhibited mineral loss, now again specifically acknowledged as inhibiting bone resorption.

An additional* ex vivo* experiment, which did include some PPi dosing, was published a year later. It was reported [5] that PPi at 4 *µ*gms P/mL (the interesting very low dose from the embryonic chick femur model was either not tried or not reported) did not inhibit the release of labeled calcium from cultured neonatal mice calvaria. Such an inhibition was seen with BPs. This paper included the statement that “in living systems, diphosphonates decrease bone resorption… where PPi is ineffective.” The proviso mentioned above that PPi destruction through hydrolysis might explain the observation failure was again included.

### 3.3. A Pivotal* In Vivo* Model

In the same publication as the chick calvaria experiments, the effects reported in the 1968 short communication [14] of PPi and BPs on thyroparathyroidectomized rats were described in detail. In this living model, administration of parathyroid hormone (PTH) was known to effect a rise in plasma calcium. Animals pretreated for 3 days with subcutaneous PPi (as well as a number of other phosphates) did not differ from controls; the PTH-induced rise in plasma calcium persisted. A conclusion was reached that PPi did not block the effect of PTH. However, this subcutaneous pretreatment was* not continued* on the day the PTH effect was measured. This is surprising, since parallel experiments [17] in the same lab around the same time had demonstrated the short-lived persistence of exogenously administered PPi. The BPs, which were stable after injection, induced the expected blockage of the rise in calcium despite the interval between pretreatment and PTH challenge.

From this date, a conviction apparently arose that PPi could not be shown to regulate bone resorption.

Evidence had been accumulating [18] that PTH release of calcium from living bone was cell-mediated. An additional contribution (see below) to this understanding was described in the publications of one of us. At about that same time, the Fleisch group began to tacitly acknowledge [5] that the theoretical physical chemical inhibition of dissolution of HAp by BPs as an explanation of PTH-stimulated calcium release should be replaced by the inhibition of a cell-mediated process. Understanding the cell-mediated nature of bone resorption permits realization that the “small increase in mineral deposition” seen with very low dose PPi is in fact a decrease in net mineral lost due to resorption, which is, in the controls, exceeding the resorption in the presence of PPi. This explanation was not offered nor, apparently, recognized.

Russell continued to publish collaboratively with Fleisch until at least 1975 [19]. In that year they summarized progress in the development of the BPs as having “effects similar to PPi on calcium phosphates* in vitro,* but to be* more* [our emphasis] potent in inhibiting… resorption of bone in a variety of experimental living systems.” This implies that dual physiologic properties of PPi were at least suspected, but attention from this time was focused on the development of the BPs. This implied concession of possible antiresorptive effects of PPi is consistent with the statement on the present BP labels mentioned above. It is, however, inconsistent with the recent [4] categorical denial of such a role.

Fleisch had already reported [17] animal studies of intravenously administered ^32^P-labeled PPi in a paper recently reviewed [4] with justifiable brevity, however newly enhanced by a large and colorful illustrative figure. The Fleisch study described the experimentally determined fate of injected labeled PPi as represented by a two-compartment partition equation with rapid disappearance from the first compartment. In retrospect, it seems very difficult to imagine that the second compartment was not acknowledged to be bone, although the last few lines suggest that the “anatomical identity” was consistent with either “soft tissues or bone.” Indeed, the recent review adds a (symbolic?) bone icon near that compartment illustrated by the colorful figure.

In any case, three years later the group published an affinity study [20] showing where the PPi relocated. When incubated with HAp suspension, an initially rapid disappearance of PPi from solution was observed. The rate of disappearance tapered off but continued at a much slower rate for at least several days. This binding was temperature and pH dependent, maximizing near the physiologic norms. Two BPs were described with similar binding behavior. The binding was competitive and one of the BPs, ethane-1-hydroxy-1,1-diphosphonate (EHDP), was more effective at displacing PPi than the reverse. Orthophosphate was released into solution [21]. This Fleisch HAp affinity paper also referenced an earlier Rasmussen publication of an intravenous thyroparathyroidectomized rat protocol using simultaneously initiated infusions of PTH and PPi [22]. This Rasmussen citation was acknowledged to suggest that PPi “may decrease bone resorption when administered parenterally.” The Fleisch paper ignored the subsequent PTH/PPi sequential-initiation Delong et al. protocol [6] which had been published two years earlier in the same journal. However, additional work suggestive of antiresorption by a Japanese group was cited (see below).

To recapitulate, Fleisch neither provided much evidence for, nor proved a lack of, an antiresorptive effect associated with PPi. The record shows that he expected such an effect. He looked moderately carefully for it but concluded, to be precise, that subcutaneously administered PPi was insufficiently stable in living systems to block PTH-induced resorption 24 hours after it was administered. This conclusion was consistent with the experimental protocol employed, but that protocol did not take any measures to circumvent the short life of exogenous PPi, which had already been demonstrated in the same laboratory.

### 3.4. Alternative Studies Consistent with PPi Antiresorptive Activity

In the early 1970s, one of the present authors (AFD) completed a series of experiments with results consistent with an antiresorptive role of PPi in a cell-mediated increase in calcium excretion effected by intravenous PTH in the thyroparathyroidectomized rat model. The protocol differed from the earlier trials in the Fleisch laboratories utilizing subcutaneous pretreatment of the rats a day before PTH challenge. After testing [22] simultaneous intravenous infusion of PTH + PPi, it was found that beginning the PPi infusion prior to the PTH challenge allowed the PPi suppression of calciuria to be better demonstrated. Control animals were challenged separately with PPi or PTH. In these controls, PTH caused an immediate rise in urinary orthophosphate but first invoked a slight drop in urinary calcium and then a progressive rise in urinary calcium and hydroxyproline excretion. Hydroxyproline was recognized [23, 24] at the time as a marker of bone resorption. The delay in calcium excretion was attributed to the direct effect of PTH increasing renal calcium retention, an effect now well established [25].

When pyrophosphate at pH 7.4 was infused at 15 *µ*mols/hr for 4 hours followed by PTH challenge infused at 5 *µ*gms/hr, the rise in urinary excretion of calcium and hydroxyproline seen in the control was blocked [5]. After the cessation of PPi infusion, continued PTH was accompanied by the same rise observed in the controls. This was* exactly* the same effect that Fleisch claimed as indicative of the antiresorptive potential of the BPs. (There could not have been, of course, any return to PTH-induced calcium release when the BP administration was discontinued, since the BPs were immune to rapid hydrolysis.) Antiresorption was the interpretation that Fleisch assigned to this effect; the infusion model shows that this interpretation applies equally to PPi.

In our (AFD) experiments, the rate of urine production was not affected by PPi. The inulin clearance was used to measure the GFR, which also was not significantly affected by PPi. In the serum, PTH of course invoked a rise in calcium and orthophosphate. PPi alone also invoked a significant rise in calcium and orthophosphate in this thyroparathyroidectomized model in contrast to the effect in intact rats described below.

This protocol compensated for the rapid peripheral hydrolysis of PPi (phosphatase-mediated) by presenting sufficient inhibitor to overcome the hydrolysis effect, bind to HAp surfaces, survive local hydrolysis in the bone compartment, and exert the antiresorptive effect seen at very low levels in the earlier chick femur experiment of Fleisch.

Japanese investigators, led by Orimo, currently the President of the Japan Osteoporosis Foundation, anticipated the Rasmussen group findings in an alternative parallel preparation [26]. In the intact rat, subcutaneous calcitonin and PPi alone each induced hypocalcemia. But PPi added to the maximal effective dose of calcitonin induced an additional drop in calcium. Subcutaneous injection was used, but in this case the effect of the treatment was detectable within 30 minutes. Significantly, serum calcium returned to baseline within 5 hours, waiting until the next day would have missed the effect. It is evident that this happened in the Fleisch subcutaneous* in vivo* experiment described above [15].

In another paper [27], Orimo injected pregnant mice with labeled ^45^Ca to obtain labeled newborn calvaria. These were attached to coverslips by clotted chicken plasma and covered with a physiologic solution which was renewed and oxygenated every other day for a week. Aliquots of the medium were counted at each exchange by scintillation. PTH increased the release of the label and this release was inhibited by PPi at 2–16 *µ*M concentrations in the tube in a dose-dependent manner. Thyrocalcitonin (TC) inhibited the release to a somewhat greater extent. Combined PPi and TC were more inhibitory than either of the agents alone. The very similar (see above) labeled calcium preparation [5] reported in 1972 by Fleisch measured only the spontaneous release of labeled calcium. No PTH-stimulated release was studied. In that preparation, the spontaneous release in the presence of 4 *µ*gms P/mL of PPi was the same as that from live controls, whereas, in the presence of a similar concentration of each of two BPs, the release from live samples was suppressed significantly, almost to the baseline degree that was observed after killing the preparation by freezing. Thus, the degree of remodeling suppression with the bisphosphonates was very high. The design of this experiment missed any opportunity to find that PPi might function as an antiresorptive agent. PPi was not reported as being tested at the unique 1 *µ*gm P/mL which was consistent with antiresorptive activity in the original fetal chick femur model [13] from the same lab.

### 3.5. Potential Implications of Ignoring the Antiresorptive Effects of PPi

Aside from correcting the disregard of the evidence of the antiresorptive properties of PPI, from the early Fleisch chick femur experiment as well as the discoveries from the labs of Rasmussen and Orimo, why should we be concerned with rectifying such an oversight at this late date? After all, PPi is not (see below) a suitable candidate to replace the BPs as powerful and long-lasting antiresorptive agents. The Japanese group did assert long ago that PPi and calcitonin might each find a place as reversible antiremodeling agents and it is true [28] that calcitonin, despite being short-acting, was eventually approved for that and other indications. But there are at least three reasons why the recognition of dual role of PPi might have provided additional insight affecting the development and exploitation of the BP drugs.

Russell and Fleisch investigated [29] the possible place of abnormal PPi metabolism in several uncommon inherited errors of metabolism. PPi plasma levels were compared in normal subjects and in individuals with hypophosphatasia, osteogenesis imperfecta, osteopetrosis, primary hyperparathyroidism, and early onset osteoporosis.

The pooled results from 57 normal individuals gave a serum PPi average of 3.50 *µ*mol/L and a range of 0.074–0.350 *µ*gm P/mL. In 17 patients with hypophosphatasia, a group of genetic syndromes characterized by low alkaline phosphatase and contemporaneously reported [30] to suffer from “long-bone fractures” (see below for complete characterization of the fractures), the PPi levels were presented graphically in the range of 6.3–17.5 *µ*mol/L and described as “invariably above normal.” They speculated that the elevated PPi might be “the cause of the defective mineralization of bone in this disease.”

The levels of PPi in the affected individuals were mostly 2-3 times the determined normal. This (roughly 1 *µ*gm P/mL) is similar to that in the embryonic mouse femur [13] which was associated with* decreased* bone resorption not decreased mineralization, as explained above. In other words, the evidence available did not suggest a concentration sufficient to affect mineralization, but rather an antiresorptive effect, had the latter been recognized at the time of the mouse femur studies. Doubtless mineralization is defective in hypophosphatasia, but the serum PPi did not qualify as the primary cause.

It was more than 4 decades later that a similarity between fractures in hypophosphatasia [31] and BP-associated fractures was noted, first in the recognition [32] that the fractures commonly seen in hypophosphatasia resembled those increasingly reported since 2003 as associated with BP therapy and finally in the discovery [33] of a case of unsuspected subclinical hypophosphatasia which was unmasked by BP therapy, producing an additive effect on bone fragility. This finding is, at least, consistent with the prevailing continuously resupplied excess of plasma PPi in affected individuals resembling that of the antiresorptive hydrolytically stable BPs in unaffected individuals. There is, in any case, persuasive evidence [34] that subclinical hypophosphatasia is not the source of the BP-associated femur fractures.

Russell and Fleisch also found PPi in 11 patients with osteogenesis imperfecta (OI), ranging in age from ~1 to 68 (presented graphically) to be in the normal range, in contrast to a contemporaneous report [35] of elevated serum and urine levels, with the lead author of that latter report having also studied in the Labs of Neuman. This disagreement is probably resolved by the finding [36] (which was presented as a resolution of the disagreement) that PPi can be normal or elevated in individual patients with OI, possibly related [37] to the degree of disease severity. Some fluctuation between normal and elevated levels was also reported in several individuals.

The use of the BP pamidronate in OI is accepted therapy and may reduce the incidence of pathologic fractures [38]. However, again after the increasing recognition of BP-associated fractures in osteoporosis therapy, similarities have been noted in new fracture pathology in OI [39] in femurs of patients given pamidronate that have already been rodded for other reasons associated with OI. These fractures have not been associated with stress riser locations near the ends of the rods but conform to the usual BP-associated characteristics in spite of the rods. Levels of PPi in the affected individuals have not been reported, but it is possible that an additive effect is being detected. Periprosthetic fractures characteristics of the BP-associated atypical femoral fractures have also been reported [40] in patients without OI. These fractures do not appear to be as distinct from stress riser effects as those reported in OI.

Thirdly, there is persuasive legacy evidence [41–45] that workers in the phosphorus match industry exposed chronically to inhalation of the fresh smoke of burning white phosphorus developed incapacitating ailments resembling the jaw osteonecrosis and midfemoral fractures recognized [46] as associated with the BP drugs. The major constituent of this smoke is known to be PPi [47]. This implies that had PPi been developed as a daily injectable or nasally administered osteoporosis treatment like calcitonin, it might well have exhibited the same serious side effects as the BPs.

## 4. Conclusions

In summary, we have reviewed the evidence that pyrophosphate has antiresorptive properties when present in the serum at concentrations slightly above normal. This evidence was ignored and the property was misleadingly denied in a recent review [4]. Confirmation can be found in the low PPi dose example of the chick embryo study of Fleisch and in the* in vivo* protocols of Rasmussen and Orimo.

We have also shown that this legacy misapprehension can and probably has had serious implications regarding the use of BP therapy. Attention to the evaluation of PPi levels in affected OI individuals who have incurred such fractures might prove useful in defining those most susceptible to the reported complications.

The several antiosteoporosis drugs that operate through natural physiologic receptors are structurally analogous mimetics of 17*β*-estradiol, parathyroid hormone, thyrocalcitonin, and parathyroid hormone related protein. These drugs all have predictable side effects, but their design exploits pharmacologic activation of natural physiologic pathways (including both antiresorptive and anabolic examples) resulting in predictable skeletal benefits. This approach, which avoids nonphysiologic consequences, can be contrasted with pharmacodynamic modification of results near or at the end of physiologic sequences, which may result in a specific measurable change but should be understood as potentially more likely to be associated with unanticipated consequences. The management of bone mineral density loss by the reduction of bone remodeling can result in a conveniently measurable increase in bone mineral density but also risks concomitant interference with the natural processes of maintenance of skeletal integrity. The oral and intravenous BP drugs are examples of the latter nonphysiologic approach. Other intermediate-term agents effecting the same result by other means are also prescribed. Recognition of the antiresorptive effects of pyrophosphate in the “natural experiments” described above might have helped avoid some of the consequences of long term remodeling inhibition now being encountered.

In contrast, the antiresorptive drugs operating through the natural receptors provide a modulation more due to the “built-in” feedback effects of the stimulated pathway than to the characteristics of the specific remedy [48]. This commends their utilization as alternative treatments for increased bone fragility.

## Figures and Tables

**Figure 2:**